# Interaction Analysis of lncRNA and mRNA Based on the Full-Length Transcriptome of the Nymph-to-Adult Developmental Transition of *Sogatella furcifera*

**DOI:** 10.3390/insects14040308

**Published:** 2023-03-23

**Authors:** Zeyan Jia, Xibin Yang, Hong Yang, Renhuai Dai, Qinghui Zeng, Daochao Jin

**Affiliations:** Guizhou Provincial Key Laboratory for Agricultural Pest Management of Mountainous Regions, Scientific Observing and Experimental Station of Crop Pests in Guiyang, Ministry of Agriculture and Rural Affairs, Institute of Entomology, Guizhou University, Guiyang 550025, China

**Keywords:** *Sogatella furcifera*, long noncoding RNAs, molting process, temporal expression, hub lncRNA

## Abstract

**Simple Summary:**

Long noncoding RNAs (lncRNAs) have been reported to be involved in multiple biological processes. However, how lncRNA and mRNA cooperatively participate in regulating the molting process remains unknown. In this study, we constructed the full-length transcriptome of the nymph-to-adult developmental transition of *Sogatella furcifera* (white-backed planthopper) and identified three key lncRNAs: *MSTRG.16086.1*, *MSTRG.16087.1*, and *MSTRG.2447.1*, which may be involved in specific molting process of *S. furcifera*. Our results revealed that lncRNA may play a potential regulatory role and provide data for supporting further research on the molecular mechanism by which lncRNA and target genes regulate the molting of *S. furcifera*.

**Abstract:**

Little is known on how long noncoding RNAs (lncRNAs) and mRNAs cooperatively participate in regulating the nymph-to-adult development transition of *Sogatella furcifera*. Herein, lncRNA and mRNA libraries were constructed in three different developmental stages of *S. furcifera*, namely, prior to (PE), during (DE), and after (AE) ecdysis. Overall, 4649 lncRNAs were identified and divided into intergenic (53.90%), intronic (1.33%), sense (8.99%), antisense (21.75%), and bidirectional (3.94%) lncRNAs. Moreover, 795 differentially expressed lncRNAs were identified. Specifically, upon comparing PE and DE, 2719 target mRNAs were predicted for 574 lncRNAs. Upon comparing PE and AE, 2816 target mRNAs were predicted for 627 lncRNAs. Finally, upon comparing DE and AE, 51 target mRNAs were predicted for 35 lncRNAs. Kyoto Encyclopedia of Genes and Genome functional enrichment analysis revealed that the target genes of 795 lncRNAs were enriched in metabolic pathways, amino sugar and nucleotide sugar metabolism, and fatty acid metabolism. Subsequently, interaction analysis indicated that *MSTRG.16086.1*, *MSTRG.16087.1*, and *MSTRG.2447.1* were functionally associated with cuticle protein and chitin biosynthesis. Finally, 11 differentially expressed lncRNAs were significantly enriched in 3rd and 4th instar nymphs. Our findings suggest that lncRNAs play a critical regulatory role during the molting of *S. furcifera*.

## 1. Introduction

Noncoding RNAs (ncRNAs), including ribosomal RNAs (rRNAs), transfer RNAs (tRNAs), small ncRNAs (snRNAs) with a nucleotide length <200, and long ncRNAs (lncRNAs) with a nucleotide length >200, are widely found in organisms [1,2]. lncRNAs have no apparent protein-coding potential [3], are transcribed by RNA polymerase II or III, and are 5′-end-capped and 3′-end-tailed, with short transcript length and low expression [4,5]. Based on the relative positions and transcriptional directions of lncRNAs and protein-coding genes, lncRNAs are classified into four categories: sense, antisense, intronic, and intergenic lncRNAs [6]. Previous studies have shown that lncRNAs are important regulators in various biological processes and can regulate gene expression at the chromosomal, chromatin, transcriptional, and post-transcriptional levels [7,8,9,10].

Transcriptome sequencing is an effective method for investigating the important functions of lncRNAs, including growth and development [11,12], reproduction [13,14], and insecticide resistance [15]. For example, lincRNA-IBIN acts as a link between innate immune responses and metabolism in *Drosophila melanogaster* [16], and lncRNAs rapidly respond to diverse stimuli or stress factors and may be involved in insecticide resistance by regulating the detoxification or cuticle protein genes in *Bactrocera dorsalis* [17] and *Plutella xylostella* [18]. In *Sogatella furcifera*, lncRNAs were identified in embryos, eggs, nymphs, and adults [19,20]; further analysis revealed a high proportion of upregulated lncRNAs in embryonic, 4th and 5th instar nymphs [20]. However, the involvement of lncRNAs in the regulation of specific developmental processes of *S. furcifera* has not yet been reported.

The white backed planthopper, *S. furcifera* (Horváth) (Hemiptera: Delphacidae), is one of the most important rice pests [21]. To obtain more information about the lncRNAs associated with the molting process of *S. furcifera*, lncRNA and mRNA libraries were constructed during the molting process using same sequencing methods and different bioinformatics analyses. Subsequently, differentially expressed lncRNAs were selected for temporal and spatial analyses, and Gene Ontology (GO) and Kyoto Encyclopedia of Genes and Genomes (KEGG) enrichment analyses of the target genes of lncRNAs were performed. Finally, we conducted an interaction analysis of lncRNAs and mRNAs. The findings of this study will greatly enrich the molecular data of *S. furcifera* lncRNAs and will also support future research on the mechanism underlying the lncRNA-mediated regulation of the molting process.

## 2. Materials and Methods

### 2.1. Insect Rearing, Sample Collection, and RNA Extraction

*S. furcifera* was obtained from a rice field in Huaxi, Guiyang, Guizhou, China, in 2013 and was reared on Taichung Native 1 rice in our laboratory under specific environmental conditions. The feeding conditions were as follows: temperature, 25 °C ± 1 °C; relative humidity, 70% ± 5%; and photoperiod, 16 h of light and 8 h of darkness. The sequencing samples were collected prior to ecdysis (PE), during ecdysis (DE), and after ecdysis (AE), and each developmental time point was represented by three biological replicate samples.

Total RNA was extracted from the samples using a TRIzol reagent kit (Invitrogen, Carlsbad, CA, USA) according to the manufacturer’s protocol. RNA integrity was assessed using 1% agarose gel electrophoresis, and its concentration and purity were determined using a Nanodrop 2000 spectrophotometer (Thermo Fisher Scientific, Wilmington, DE, USA). RNA samples with adequate concentration and purity were stored in a refrigerator at −80 °C until further use.

### 2.2. Library Preparation and Sequencing Data Analysis

Overall, 1 μg of total RNA was used for sequencing. First, magnetic beads with Oligo (dT) were used to enrich eukaryotic mRNAs, and rRNA was extracted using a Ribo-Zero Magnetic Kit (Epicentre, Madison, WI, USA). Subsequently, the enriched mRNAs were fragmented using fragmentation buffer, and the first cDNA strand was synthesized via reverse transcription using random primers. The second strand of cDNA was synthesized using buffer, dNTPs, RNase H, and DNA polymerase I. A QiaQuick PCR kit (Qiagen, Venlo, The Netherlands) was used to purify cDNA, perform end repair, and add the poly (A) base and sequencing adapter. The ligation products were amplified using PCR, and the target fragments were screened via agarose gel electrophoresis. Finally, sequencing was performed using Illumina HiSeq 2500 (Gene Denovo Biotechnology, Guangzhou, China) (Figure S1).

To obtain high quality clean reads, low-quality reads were removed from raw reads using Fastp version 0.18.0 [22]. After filtering clean reads, the short read alignment tool Bowtie2 version 2.2.8 [23] was used to locate the reads within the ribosome RNA database. Without allowing mismatches, we removed reads from the alignment and retained unmapped reads. HISAT2 version 2.1.0 [24] was used to compare transcripts with the reference genome of *S. furcifera* (PRJNA331022) [25]. Finally, according to a reference-based approach, String Tie version 1.3.4 [26,27] was used to assemble mapped reads for each sample and calculate the transcript fragment value per kilobase per million read maps (FPKM) for each transcription region.

### 2.3. Identification of lncRNAs

Raw data were free of low-quality reads, and the clean data were mapped to the reference genome of *S. furcifera* and were divided into twelve categories using Cuffcompare. Transcripts with one of the class codes “u, i, j, x, c, e, o” were defined as novel transcripts. Coding-Non-Coding Index (CNCI) version 2.0 [28] and Coding Potential Calculator (CPC) version 0.9-r2 [29] were used to predict the coding ability of new transcripts, and the intersection of transcripts with no coding potential was used as the newly predicted lncRNA. Based on the transcription position and direction of lncRNAs and protein-coding genes, the types of lncRNAs were determined; moreover, the transcript and open reading frame (ORF) lengths and expression abundance of lncRNAs and mRNAs were compared and analyzed.

### 2.4. Analysis of Differentially Expressed lncRNAs and Prediction of Target Genes

To further analyze the differentially expressed lncRNAs involved in the molting process of *S. furcifera*, String Tie 1.3.4 was used to calculate the FPKM values of lncRNAs in each sample. Differential expression analysis was performed using DESeq2 [30], and a false discovery rate of <0.05 and |log2FC (Fold Change)| of ≥2 indicated differentially expressed transcripts.

LncRNA target genes were predicted according to their colocation (cis) and co-expression (trans) with protein-coding genes. According to the principle of cis-action prediction, the function of lncRNA was related to adjacent protein-coding genes, and the target genes located within 10 kb upstream or downstream of the lncRNA were considered as cis target genes. For the trans-lncRNAs, the expression of differentially expressed lncRNAs and protein-coding genes was used to analyze the co-expression relationships between them. An absolute value of Pearson’s coefficient |r| of >0.95 indicated co-expression. Subsequently, we used the same analysis to map differential mRNAs, cis and trans target genes of differential lncRNAs in the molting process of *S. furcifera* to the GO (http://www.geneontology.org/ (accessed on 26 April 2019)) and KEGG [31] pathway, and applied hypergeometric tests to identify the differential genes involved in GO entries and KEGG pathway.

### 2.5. Expression Dynamic Analysis of lncRNAs and mRNAs

To examine the expression patterns of differentially expressed genes in the molting process of *S. furcifera*, the expression data of each sample were normalized to 0, log2(v1/v0), and log2(v2/v0). Subsequently, Short Time-series expression Miner (STEM) version 1.3.11 [32] was used to analyze the expression patterns of differentially expressed genes.

### 2.6. Interaction Analysis between lncRNAs and mRNAs

Pearson’s correlations between differentially expressed lncRNA and mRNA were determined using R software, and |r| values of >0.98 indicated co-expression. Furthermore, Cytoscape version 3.6.1 was used to construct a co-expression network.

### 2.7. RT–qPCR Analysis

To clarify the spatial and temporal expression characteristics of differentially expressed lncRNAs, we randomly selected 11 differentially expressed lncRNAs for performing RT–qPCR. The sampling times of different developmental stages were as follows: 1st instar on days 1 and 2; 2nd instar on days 1 and 2; 3rd instar on days 1, 2, and 3; 4th instar on days 1, 2, and 3; and 5th instar on days 1, 2, and 3. Different tissue samples were randomly dissected from the head, integument, foot, fat body, and gut of 5th instar nymphs. All samples were set up with three biological replicates. Total RNA was extracted using an HP Total RNA Kit (Omega, Bio-Tek, Norcross, GA, USA).

LncRNA reverse transcription was performed as follows. Briefly, 1 μg of RNA was transcribed into cDNA using lnRcute lncRNA First-Strand cDNA Synthesis Kit (Tiangen Biotech, Beijing, China). RT–qPCR was performed using lnRcute lncRNA qPCR Kit (SYBR Green) (Tiangen). qPCR was performed in a final volume of 20 µL containing 2 µL of the sample cDNA, 1 µL of each forward and reverse primer (10 µM), 6 µL of RNase-free water, and 10 µL of 2X lnR lncRNA PreMix. The PCR conditions were as follows: predeformation at 95 °C for 3 min, followed by 40 cycles of amplification (95 °C for 5 s and 60 °C for 15 s) and melting curve analysis at 65 °C–95 °C.

mRNA reverse transcription was performed as follows. Briefly, 1 μg of RNA was reverse-transcribed into the first-strand cDNA template using PrimeScript^TM^ RT Reagent Kit with gDNA Eraser (Takara, Dalian, China). RT–qPCR was performed using FastStart Essential DNA Green Master. All reactions were performed using the CFX96 Real-Time System (Bio-Rad, Hercules, CA, USA) instrument. The ribosomal protein L9 (*Sf RPL9*, GenBank accession number: KM885285) genes of *S. furcifera* were used as internal controls. All RT–qPCR primers were in line with the amplification efficiency. All lncRNA and mRNA primers are listed in Table S1.

### 2.8. Statistical Analysis

The 2^−∆∆Ct^ method [33] was used to calculate the relative expression of lncRNA and mRNA. Then, using one-way ANOVA followed by Tukey’s multiple test (*p <* 0.05) using SPSS 22.0 statistical software (IBM Corp., Armonk, NY, USA). GraphPad Prism 8.0.1 software was used for graphing.

## 3. Results

### 3.1. Identification and Characterization of lncRNAs in S. furcifera

**Figure 1:**
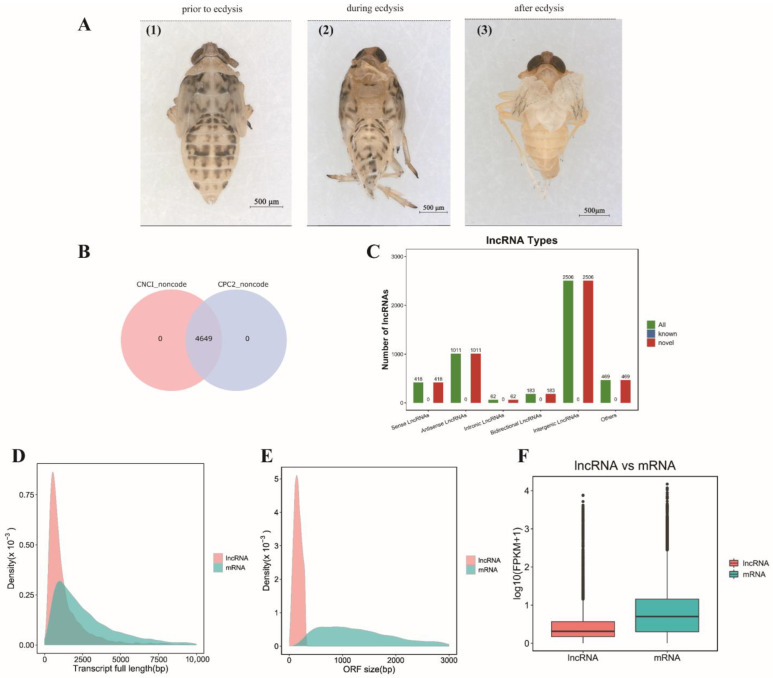
Identification of lncRNAs in *S. furcifera*. (**A**) Collection of samples for RNA-seq analysis during the nymph-to-adult transition of *S. furcifera*, (1) prior to ecdysis (PE), (2) during ecdysis (DE), (3) after ecdysis (AE); (**B**) lncRNAs predicted using two softwares (CNCI and CPC2); (**C**) classification of identified lncRNAs; (**D**) full-length transcript distribution; (**E**) maximum open reading frame (ORF) length distribution; (**F**) expression analysis of lncRNAs and mRNAs.

To systematically identify the lncRNAs associated with the molting process of *S. furcifera*, sample libraries were constructed at three developmental time points (Figure 1A) using the Illumina HiSeq 2500 platform. The original data of nine libraries obtained via sequencing were uploaded to NCBI (PRJNA916344). After filtering low-quality reads and poly A and adapter sequences, 243005728, 248384402, and 259589912 clean reads were obtained (Table S2). The average GC content was 47.41%, and the average Q20 and Q30 value of each sample was >97.70% and >93.88%, respectively, indicating that the sequencing quality was appropriate for subsequent analysis (Table S2). Later, clean reads were mapped to the reference genome of *S. furcifera* using HISAT2. 2.4, and the mapped rate of the nine samples was 71.03–88.38% (Table S2). The mapped reads were then assembled and quantified using String Tie.

The lncRNAs were identified from unknown transcripts based on their transcript length and coding ability. In this study, 4649 lncRNAs were identified during the molting process of *S. furcifera* (Figure 1B). Based on the position of lncRNAs in the genome relative to mRNAs, lncRNAs were classified into intergenic (2506; 53.90%), antisense (1011; 21.75%), sense (418; 8.99%), bidirectional (183; 3.94%), and intronic (62; 1.33%) lncRNAs (Figure 1C). The lengths of lncRNA transcripts were shorter than those of mRNA transcripts. In particular, the lengths of lncRNA transcripts ranged from 206 to 29,621 bp, and 80.43% of the lncRNAs contained <2000 nucleotides (Figure 1D). The ORF length of lncRNAs was shorter than that of mRNAs (Figure 1E), and the expression of lncRNAs was significantly lower than that of mRNAs (Figure 1F).

### 3.2. Analysis of Differentially Expressed lncRNAs and mRNAs

**Figure 2:**
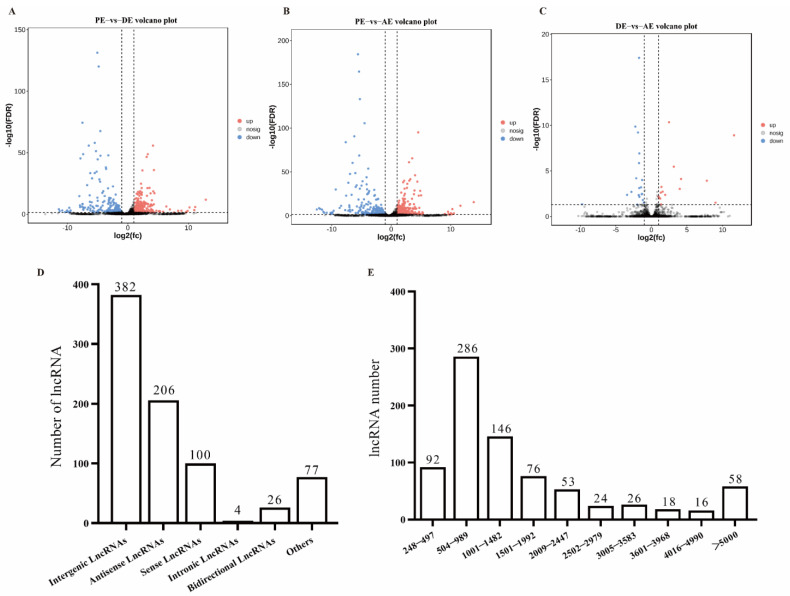
Analysis of differentially expressed lncRNAs. (**A**) Volcanic map of differentially expressed lncRNAs between PE and DE; (**B**) volcanic map of differentially expressed lncRNAs between PE and AE; (**C**) volcanic map of differentially expressed lncRNAs between DE and AE; (**D**) classification of differentially expressed lncRNAs; (**E**) transcript lengths of differentially expressed lncRNAs.

To examine the lncRNAs and mRNAs involved in the molting process of *S. furcifera*, we analyzed their differentially expressed genes. In total, 795 differentially expressed lncRNAs were identified during the molting process (Table S3). In the prior to ecdysis (PE) vs. during ecdysis (DE) comparison, 281 up- and 293 downregulated lncRNAs were identified (Figure 2A). Further, in the PE vs. after ecdysis (AE) comparison, 93 up- and 334 downregulated lncRNAs were identified (Figure 2B). Finally, in the DE vs. AE comparison, 15 up- and 20 downregulated lncRNAs were identified (Figure 2C). The results of the lncRNA classification based on transcript length revealed that the lncRNAs were classified into intergenic (382; 48.05%), antisense (206; 25.91%), sense (100; 12.58%), intronic (4; 0.50%), and bidirectional (26; 3.27%) lncRNAs (Figure 2D). The proportion of lncRNAs with a transcript length of 248–989 bp was 47.55%, that of 1001–1992 bp was 17.23%, and that of >5000 bp was 7.30% (Figure 2E).

**Figure 3:**
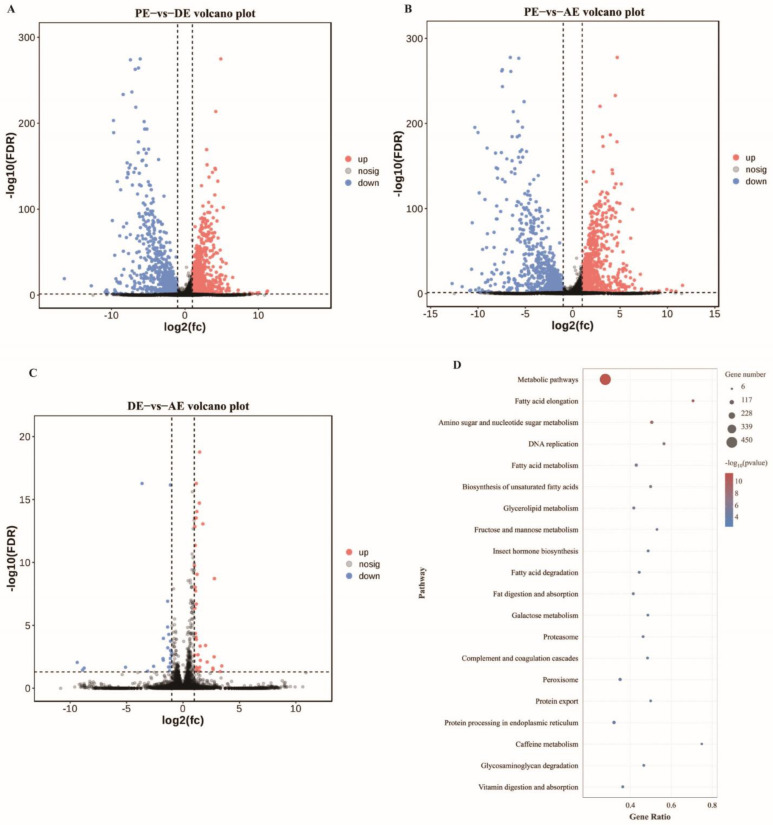
Analysis of differentially expressed mRNAs. (**A**) Volcanic map of differentially expressed lncRNAs between PE and DE; (**B**) Volcanic map of differentially expressed lncRNAs between PE and AE; (**C**) Volcanic map of differentially expressed lncRNAs between DE and AE; (**D**) The top 20 enriched KEGG pathways of the mRNAs involved in the molting process.

In total, 14,602 mRNAs were identified, of which 3543 were differentially expressed during the molting process of *S. furcifera*. In the PE vs. DE comparison, 1275 up- and 1605 downregulated mRNAs were identified (Figure 3A). Moreover, in the PE vs. AE comparison, 1222 up- and 1764 downregulated mRNAs were identified (Figure 3B). Finally, in the DE vs. AE comparison, 36 up- and 24 downregulated mRNAs were identified (Figure 3C). mRNA KEGG enrichment analysis revealed that mRNAs were significantly enriched in metabolic pathways, amino sugar and nucleotide sugar metabolism, and fatty acid metabolism (Figure 3D, Table S4).

### 3.3. Analysis of lncRNA and mRNA Expression Patterns

**Figure 4:**
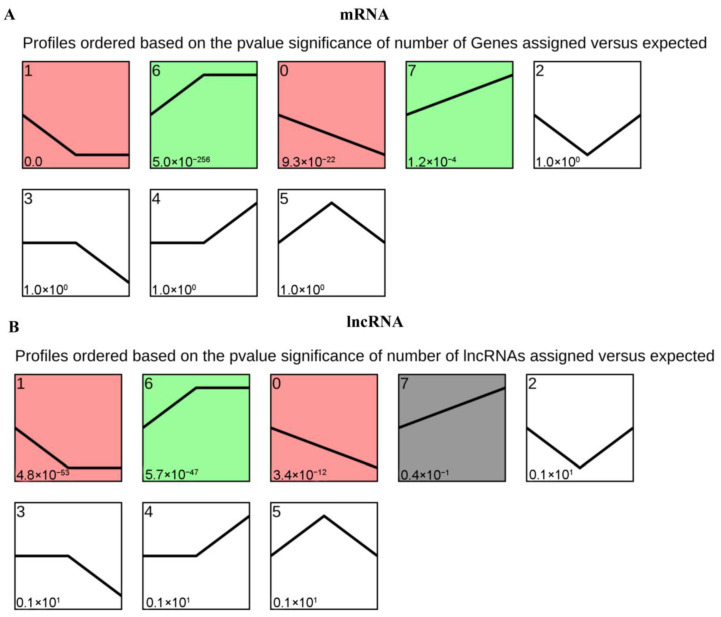
Different expression clusters of lncRNAs ad mRNAs. (**A**,**B**) The numbers in the left upper part of the boxes indicate profile IDs (0–7), whereas those in the left lower part of the boxes indicate *p*-values. The boxes are sorted by *p*-values, as calculated using a hypergeometric test, with the values presented in ascending order.

STEM software was used for cluster analysis. Filtering was performed based on the following conditions: (i) the multiple difference between the maximum and minimum gene values is less than the threshold and (ii) the correlation coefficients with all trends are lower than the threshold. Finally, 795 lncRNAs and 3543 mRNAs were converged into eight expression clusters, namely, profile zero, one, two, three, four, five, six, and seven (Figure 4). The expression of profiles six, seven, and four continued to increase, that of profiles one, zero, and three continued to decrease, and that of profile two initially decreased and then increased. Further, the expression of profile five initially tended to increase and then decreased. In total, 287 lncRNAs and 1731 mRNAs were clustered into pattern one, 270 lncRNAs and 1214 mRNAs were clustered into pattern six, 92 lncRNAs and 259 mRNAs were clustered into pattern zero, and 54 lncRNAs and 181 mRNAs were clustered into pattern seven (Table S5).

### 3.4. Analysis of the Interaction between Differentially Expressed lncRNAs and mRNAs

**Figure 5:**
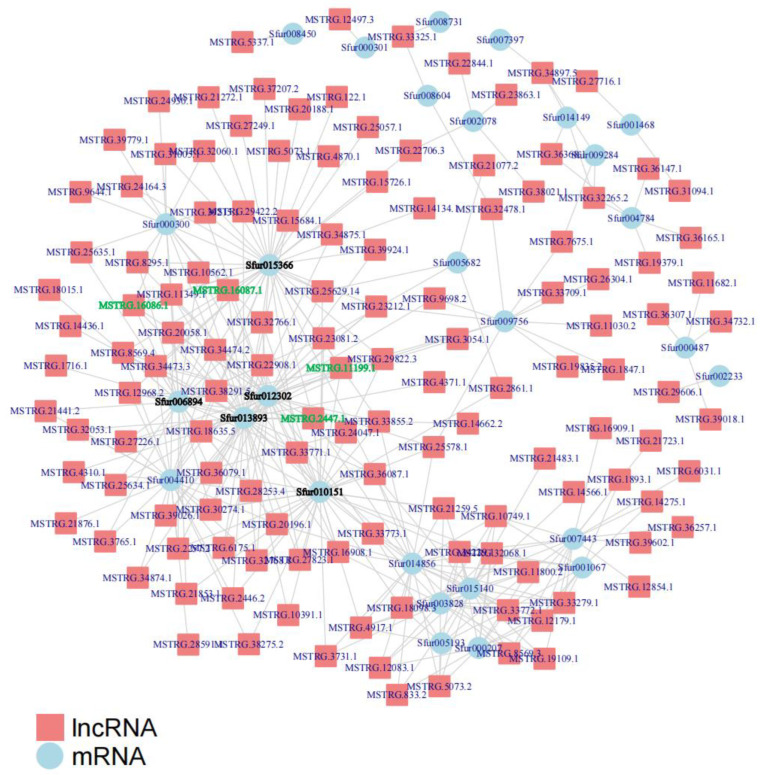
Interaction analysis of lncRNAs and protein-coding genes involved in the molting process using Pearson’s correlation coefficient.

To determine whether differentially expressed lncRNAs and mRNAs cooperatively participate during the molting process of *S. furcifera*, the potential regulatory relationship between them was analyzed via a co-expression network. As shown in Figure 5, multiple differentially expressed lncRNAs can target an mRNA. The identification and analysis of these hub mRNAs revealed that these mRNAs were involved in the growth and development of insects. Among them, several lncRNAs target the hub mRNAs sfur015366, sfur0006894, sfur013893, sfur012302, and sfur010151. Further analysis showed that these hub mRNAs encoded chitin synthase, ecdysone, cuticular protein, and fatty acid metabolism pathway genes, such as *CPAP1-K*, *GNA*, *PAGM*, *E-78*, and *ELO*. Therefore, these mRNAs may play a key role in the biological process of nymph-to-adult developmental transition of *S. furcifera*. Meanwhile, the analysis of lncRNAs that target hub mRNAs revealed that *MSTRG.11199.1*, *MSTRG.16086.1*, *MSTRG.16087.1*, and *MSTRG.2447.1* target *CPAP1-K*, *GNA*, *PAGM*, *E-78*, and *ELO* (Table S6). Based on the above results, we speculate that these lncRNAs (with an mRNA correlation coefficient of >0.98) are important candidate genes involved in the molting process of *S. furcifera* during nymph-to-adult transitions and may exert some regulatory effects on hub mRNAs involved in the molting process *S. furcifera*.

### 3.5. Prediction and Analysis of the Target Genes of Differentially Expressed lncRNAs

**Figure 6:**
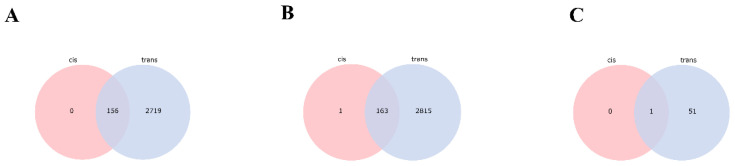
Trans/cis target gene prediction of 795 differentially expressed lncRNAs. (**A**) Venn diagram of cis/trans target genes of the PE vs. DE comparison group; (**B**) Venn diagram of cis/trans target genes of the PE vs. AE comparison group; (**C**) Venn diagram of cis/trans target genes of the DE vs. AE comparison group.

Based on the localization (cis) and co-expression (trans) of lncRNAs and protein-coding genes, the target genes of 795 differentially expressed lncRNAs involved in the molting process of *S. furcifera* were predicted. In the PE vs. DE comparison, 2719 target mRNAs (cis: 156; trans: 2875; and both cis and trans: 156) were predicted for 574 differentially expressed lncRNAs (Figure 6A). In the PE vs. AE comparison, 2816 target mRNAs (cis: 164; trans: 2978; and both cis and trans: 163) were predicted for 627 differentially expressed lncRNAs (Figure 6B). Finally, in the DE vs. AE comparison, 51 target mRNAs (cis: 1; trans: 52; and both cis and trans: 1) were predicted for 35 differentially expressed lncRNAs (Figure 6C; Table S7).

**Figure 7:**
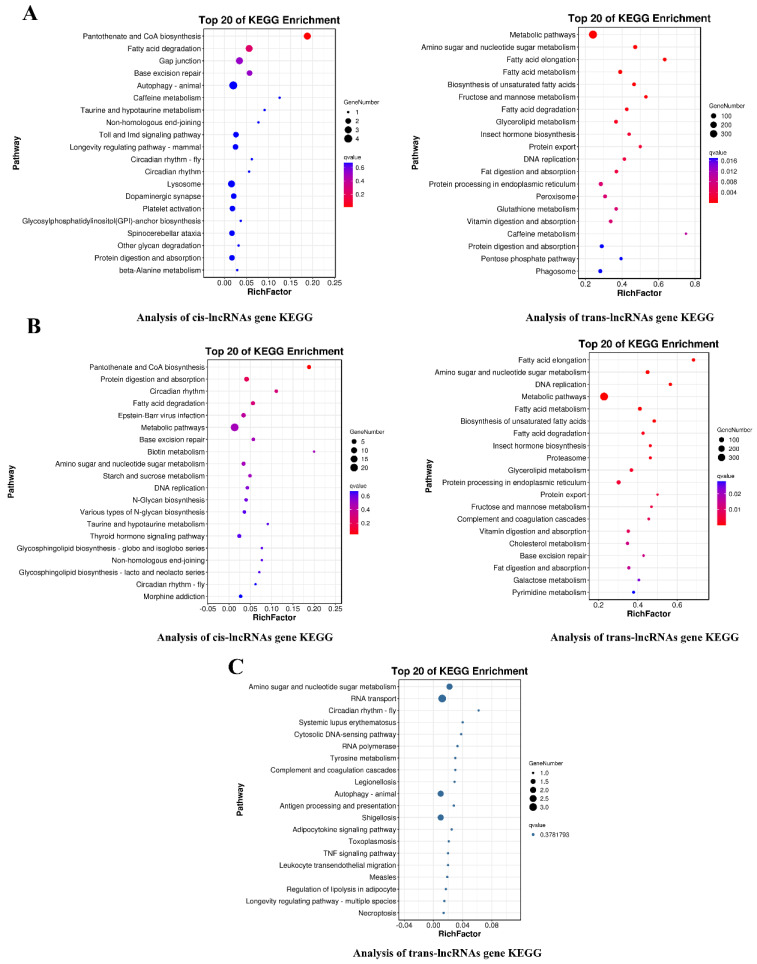
Predicted target genes of the differentially expressed lncRNAs. (**A**) KEGG enrichment analysis of PE vs. DE cis/trans lncRNA target genes. (**B**) KEGG enrichment analysis of PE vs. AE cis/trans lncRNA target genes. (**C**) KEGG enrichment analysis of DE vs. AE trans lncRNA target genes.

GO analysis revealed that the target genes of 795 differentially expressed lncRNAs were enriched in the single-organism, cellular, and metabolic processes as well as in the molecular function processes of the structural constituents of cuticle (GO: 0042302) and chitin-based cuticle (GO: 0005214) (Figure S2; Table S8). KEGG enrichment analysis revealed that these 795 cis and trans target genes of lncRNAs were enriched in metabolic pathways, amino sugar and nucleotide sugar metabolism, fatty acid metabolism, and cAMP signaling pathway (Figure 7; Table S9). In addition, based on the prediction results of target genes, we identified chitin metabolism pathway genes, such as *CHT3*, *CHT4*, *CHT5*, *CDA4*, *GFAT*, *HK1*, *CHS1*, *PAGM*, *GNA*, and *G6P1*, and these mRNAs may be regulated by *MSTRG.2447.1*, *MSTRG.16086.1*, *MSTRG.16087.1*, *MSTRG.19835.2*, and *MSTRG.8295.1* (Table S10). In conclusion, lncRNAs may be involved in specific biological processes during the molting process of *S. furcifera*.

### 3.6. Verification of Sequencing Results

**Figure 8:**
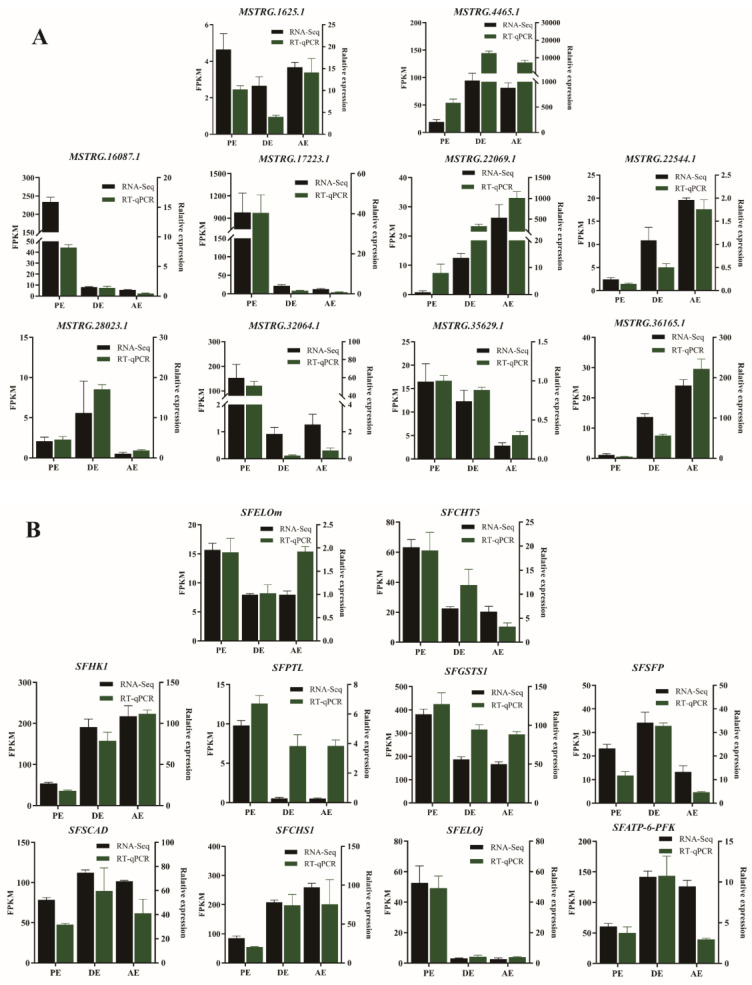
Validation of differentially expressed lncRNAs and mRNAs via RT–qPCR. (**A**) Differentially expressed lncRNA analysis; (**B**) differentially expressed mRNA analysis.

To verify the reliability of RNA-seq data, we randomly selected 10 lncRNAs and 10 mRNAs during the molting process of *S. furcifera* for RT–qPCR based on previous expression pattern analysis of differentially expressed genes. The 10 selected lncRNAs were as follows: *MSTRG.17223.1*, *MSTRG.32064.1*, *MSTRG.16087.1*, *MSTRG.35629.1*, *MSTRG.1625.1*, *MSTRG.28023.1*, *MSTRG.4465.1*, *MSTRG.22069.1*, *MSTRG.22544.1*, and *MSTRG. 36165.1*. The 10 selected mRNAs were as follows: *SFATP-6-PFK*, *SFCHS1*, *SFCHt5*, *SFELOj*, *SFELOm*, *SFGSTS1*, *SFHK1*, *SFPTL*, *SFSFP*, and *SFSCAD*. As shown in Figure 8, the RT–qPCR results of these lncRNAs and mRNAs in the three developmental stages (PE, DE, and AE) were consistent with the expression trend of high-throughput sequencing results, thereby indicating that the results of high-throughput sequencing were reliable.

### 3.7. Spatial and Temporal Expression Patterns of Differentially Expressed lncRNAs

**Figure 9:**
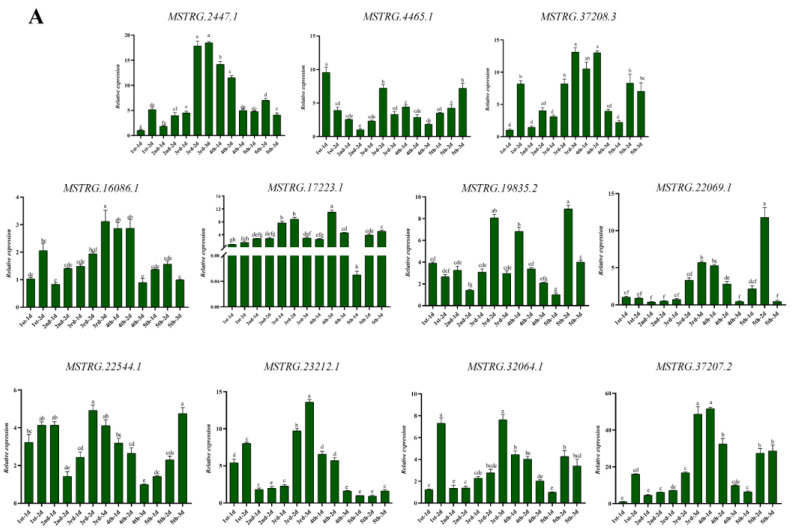

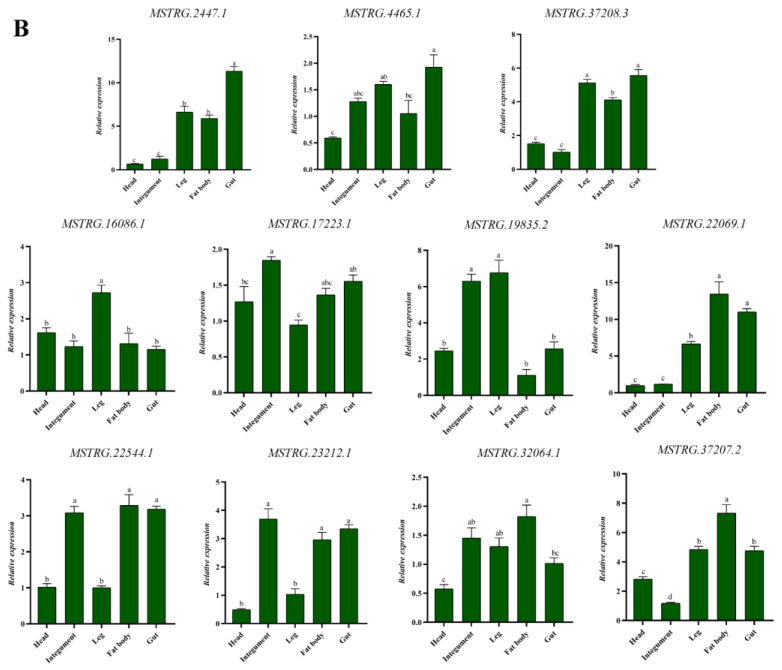
Developmental and tissue expression patterns of Profile 1 (MSTRG.37207.2, MSTRG.2447.1, MSTRG.17223.1, MSTRG.32064.1, MSTRG.37208.3, MSTRG.16086.1), Profile 6 (MSTRG.22069.1, MSTRG.4465.1, MSTRG.23212.1, MSTRG.19835.2), and MSTRG.22544.1 genes. (**A**) Expression patterns of differentially expressed lncRNAs in different developmental stages; (**B**) expression patterns of differentially expressed lncRNAs in different tissues of S. furcifera. Lowercase letters above the bars indicate significant differences (Tukey’s HSD, ANOVA, *p* < 0.05).

We selected 11 differentially expressed lncRNAs, and their expression in different developmental stages and tissues of *S. furcifera* was analyzed using RT–qPCR. RT–qPCR results revealed that 11 lncRNAs were expressed in different developmental stages and tissues of *S. furcifera*. In these developmental stages (Figure 9A), *MSTRG.2447.1*, *MSTRG.23212.1*, *MSTRG.37207.2*, *MSTRG.16086.1*, and *MSTRG.37208.3* exhibited a similar expression trend in the first to fifth instar of *S. furcifera*, with the highest expression in the third and fourth instar of *S. furcifera*. Further, *MSTRG.4465.1*, *MSTRG.32064.1*, *MSTRG.19835.2*, *MSTRG.22069.1*, *MSTRG.22544.1*, and *MSTRG.17223.1* exhibited a fluctuating expression trend of decreasing first and then increasing in the first to fifth instar of *S. furcifera*, among which the expression of *MSTRG.19835.2*, *MSTRG.22069.1*, and *MSTRG.22544.1* was significantly upregulated in the second and third day fifth instar *S. furcifera*. These findings indicate that these three lncRNAs may play an important role in this stage of development.

In different tissues (Figure 9B), *MSTRG.16086.1* was highly expressed in the foot; *MSTRG.22069.1* and *MSTRG.37207.2* showed the highest expression in the fat body; *MSTRG.2447.1* and *MSTRG.4465.1* were highly expressed in the gut; *MSTRG.22544.1* and *MSTRG.23212.1* were highly expressed in the integument, fat body, and gut; *MSTRG.17223.1* showed the highest expression in the integument; *MSTRG.19835.2* showed the highest expression in the foot and integument; *MSTRG.32064.1* showed the highest expression in the fat body; and *MSTRG.37208.3* was highly expressed in the gut and foot. Among them, *MSTRG.22544.1*, *MSTRG.23212.1*, *MSTRG.17223.1*, and *MSTRG.19835.2* were significantly expressed in the integument of *S. furcifera*.

## 4. Discussion

Molting is important for the growth and development of insects. As important transcriptional regulators, lncRNAs have been identified in various development stages of *S. furcifera* [19,20]. However, no reports on lncRNAs exist with regard to the molting process of *S. furcifera*. Therefore, to determine whether lncRNAs and mRNAs cooperatively participate in the regulation of the molting process of *S. furcifera*, lncRNA and mRNA libraries were constructed. Accordingly, 4649 lncRNAs were identified and classified into intergenic, intronic, sense, antisense, and bidirectional lncRNAs; among them, intergenic lncRNAs showed the highest proportion of lncRNAs. In previous studies [19,20], lncRNA identification was limited to the different developmental stages of *S. furcifera*. In this study, PE, DE, and AE transcriptome libraries were constructed based on the reference genome of *S. furcifera* at three specific developmental time points, thereby enriching the basic research on *S. furcifera* lncRNAs. Among insects, differentially expressed lncRNAs have been reported in *P. xylostella* [18], *Tribolium castaneum* [12], and *Panonychus citri* [34] in different developmental stages, with intergenic lncRNAs exhibiting the highest proportion, which is consistent with the results of this study. lncRNAs identified in different insects vary in proportion, and our study showed a small proportion of lncRNAs with several types. However, the proportion of intronic lncRNAs was higher than that of intergenic lncRNAs in *Aedes aegypti* [11] and *Spodoptera litura* [15]. Altogether, among the reported insect lncRNAs, the proportion and types of lncRNAs differ, with the highest proportion of intergenic lncRNAs. However, the proportion of intronic lncRNAs is higher than that of intergenic lncRNAs in some insects. In addition, the transcript and ORF lengths of lncRNAs were shorter than those of mRNAs, and the expression of lncRNAs was lower than that of mRNAs; these findings are consistent with those of other insect studies [11,35].

Differentially expressed lncRNAs were analyzed to focus on the regulation of key lncRNAs in specific developmental processes. For example, the expression of 42% of lncRNAs identified in the 27 developmental processes of *D. melanogaster* was significantly upregulated during the critical period of larval development transition [35]. Moreover, time series profile analysis of differentially expressed lncRNAs in *P. citri* revealed that 77 lncRNAs were clustered into two dynamic expression profiles, implying that these lncRNAs participate in the molting process [34]. In *S. furcifera*, the expression of lncRNAs was highly time-limited and was upregulated in the embryos, as well as fourth and fifth instars [20]. In this study, to determine the key genes involved in the molting process, a cluster analysis of differentially expressed genes and spatiotemporal expression detection revealed that the expression of *MSTRG.19835.2*, *MSTRG.22069.1*, and *MSTRG.22544.1* was significantly upregulated in the second and third day fifth instars, whereas that of *MSTRG.22544.1*, *MSTRG.23212.1*, *MSTRG.17223.1*, and *MSTRG.19835.2* was significantly upregulated in the integument of *S. furcifera*. The different expression patterns of these differentially expressed genes may be related to various biological processes, which should be investigated further.

Insect molting is a complex biological process involving the degradation and synthesis of chitin as well as the co-regulation of ecdysone and juvenile hormones. In this study, lncRNA target gene enrichment analysis and lncRNA–mRNA interaction analysis were performed to determine whether lncRNAs and mRNAs cooperatively participate in the regulation of the molting process of *S. furcifera*. GO results revealed that the target genes were significantly enriched in the molecular function processes of structural constituents of cuticle (GO: 0042302) and chitin-based cuticle (GO: 0005214). Moreover, KEGG enrichment results revealed that the target genes were enriched in metabolic pathways and amino sugar and nucleotide sugar metabolism. Further analysis showed that *MSTRG.2447.1*, *MSTRG.16086.1*, *MSTRG.16087.1*, *MSTRG.19835.2*, and *MSTRG.8295.1* targeted the chitin degradation and synthesis pathway genes, including *SFCHT3*, *SFCHT4*, *SFCHT5*, *SFCDA4*, *SFGFAT*, *SFHK1*, *SFCHS1*, *SFPAGM*, *SFGNA*, and *SFG6P1*. It is well-known that the molting process of insects is closely related to chitin metabolism. Previous laboratory studies showed that the white-backed planthopper showed abnormal molt and death after injection of double-stranded RNA of *SFCHT5*, *SFCDA4*, and *SFCHS1* [36,37,38]. Moreover, lncRNA–mRNA interaction analysis revealed that *MSTRG.11199.1*, *MSTRG.16086.1*, *MSTRG.16087.1*, and *MSTRG.2447.1* genes correlated with growth and development process related mRNAs (*CPAP1-K*, *GNA*, *PAGM*, and *E-78*) more than 0.98. In *Nilaparvata lugens* studies, the silencing of cuticular protein genes resulted in malformed endocuticle or endocuticle and exocuticle structures, low reproductive ability, and egg hatchability [39]. Our study focused on target gene annotation enrichment analysis and interaction analysis of lncRNA and mRNA during the nymph-to-adult molting of *S. furcifera* and revealed that *MSTRG.2447.1*, *MSTRG.16086.1*, and *MSTRG.16087.1* may be the key lncRNAs that play a regulatory role through mRNAs. Similarly, target gene annotation in *T. castaneum* and co-expression network analysis in *P. citri* have shown that lncRNAs can target metabolic pathways as well as cuticle protein and chitin biosynthesis during growth and development [12,34]. In conclusion, based on the results of our target gene annotation and network diagram, three key lncRNAs that may be involved in the regulation of the molting process of *S. furcifera* were identified. We hypothesize that these lncRNAs regulate the molting process of *S. furcifera* through mRNAs, but the mechanism underlying the regulation remains unclear.

## 5. Conclusions

In this study, we constructed lncRNA and mRNA libraries for specific molting processes of *S. furcifera*. lncRNA target gene annotation enrichment analysis and lncRNA–mRNA interaction analysis revealed three key lncRNAs involved in the molting process of *S. furcifera*, namely, *MSTRG.2447.1*, *MSTRG.16086.1*, and *MSTRG.16087.1*. Our results demonstrated the functions of lncRNAs in regulating the molting processes of *S. furcifera*; however, further research is needed to explore these functions.

## Data Availability

All data used/generated in this study have been included in the text and Supplementary Materials of this manuscript.

## Supplementary Materials

The following supporting information can be downloaded at: https://www.mdpi.com/article/10.3390/insects14040308/s1, Figure S1: cDNA library construction schematic by Illumina HiSeq 2500 sequencing; Figure S2: GO enrichment analysis of the target genes of different expressed lncRNAs; Table S1: Primers used for RT–qPCR; Table S2: Summary of RNA sequencing data from *S. furcifera*; Table S3: Differentially expressed lncRNAs identified in the three different developmental time points assessed; Table S4: KEGG enrichment analysis of differentially expressed mRNA during the molting process; Table S5: STEM software cluster analysis of mRNA and lncRNA expression trend results; Table S6: Co-expressed network analysis of differentially expressed lncRNAs and mRNAs; Table S7: Prediction of the target genes of differentially expressed lncRNAs; Table S8: GO enrichment analysis of target genes; Table S9: KEGG enrichment analysis of target genes; Table S10: lncRNA-based prediction of amino sugar and nucleotide sugar metabolism pathway genes.
