# Supplementary material for: Interaction Analysis of lncRNA and mRNA Based on the Full-Length Transcriptome of the Nymph-to-Adult Developmental Transition of Sogatella furcifera
Source: Insects. 2023 Mar 23;14(4):308. doi: 10.3390/insects14040308 (PMC10141146; doi:10.3390/insects14040308)
Supplement: Supplementary file 1 [file insects-14-00308-s001.zip › FigS1---FigS2.pdf]

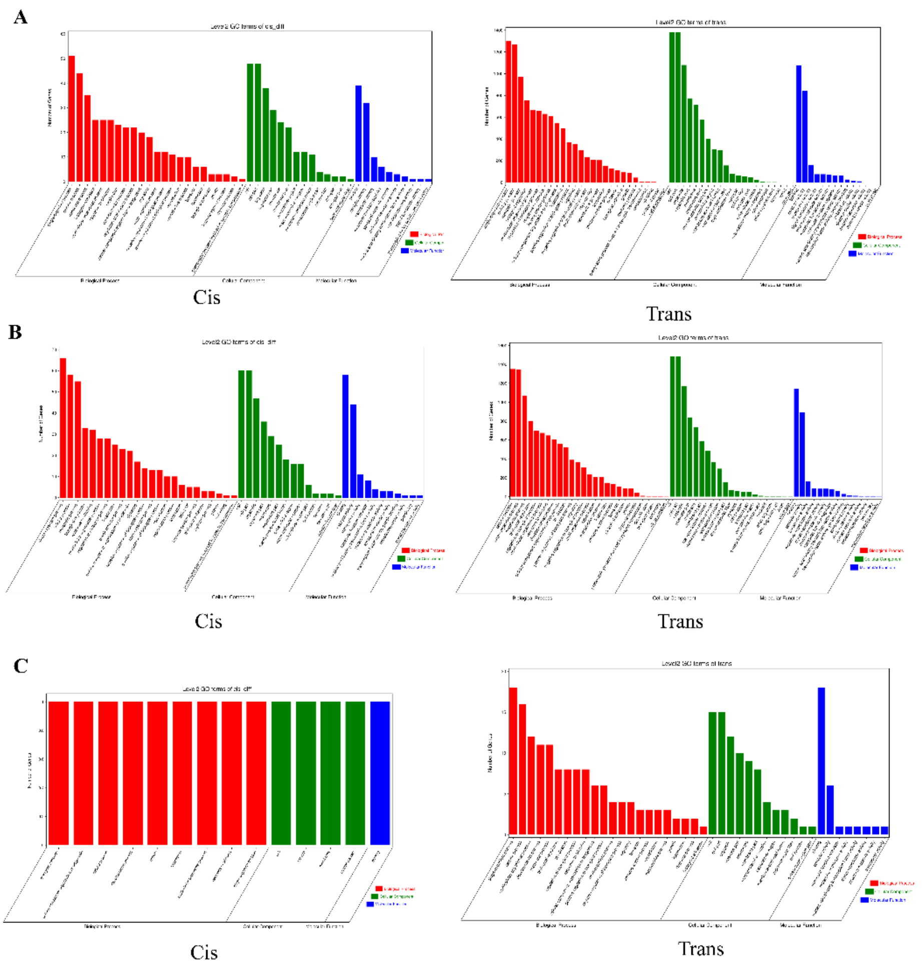

Fig S1. GO enrichment analysis of the target genes of different expressed lncRNAs. (A) PE-vs-DE cis/trans-lncRNA target gene GO enrichment analysis; (B) PE-vs-AE cis/trans-lncRNA target gene GO enrichment analysis; (C) DE-vs-AE cis/trans-lncRNA target gene GO enrichment analysis.

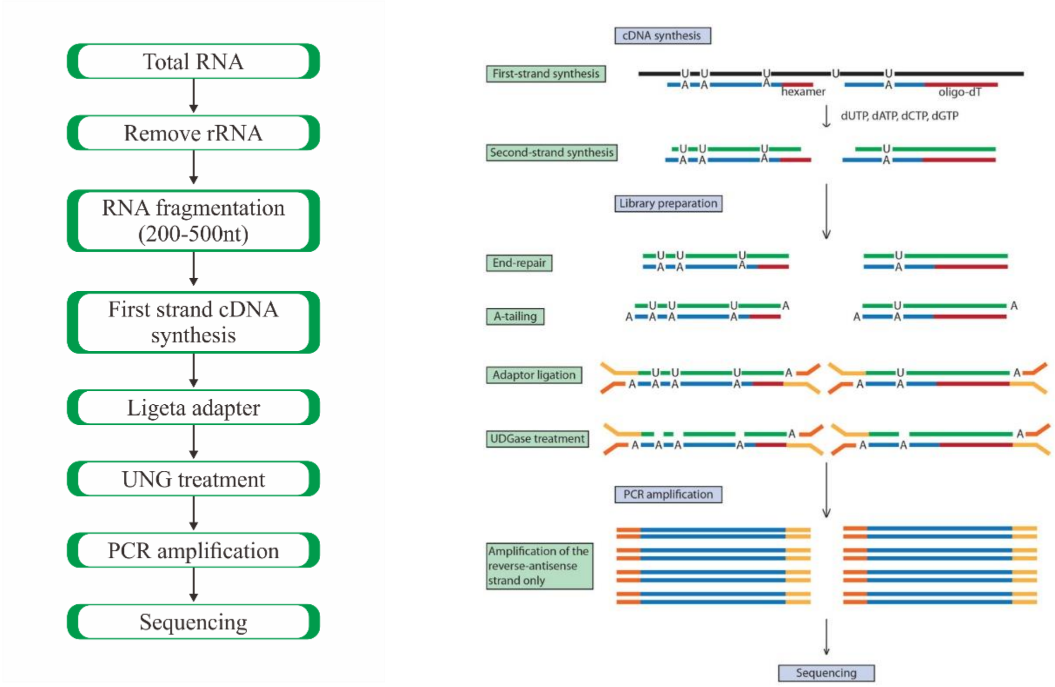

Fig S2. cDNA library construction schematic sequenced using Illumina HiSeq2500 by Gene Denovo Biotechnology Co. (Guangzhou, China)
